# Combined Input Deep Learning Pipeline for Embryo Selection for In Vitro Fertilization Using Light Microscopic Images and Additional Features

**DOI:** 10.3390/jimaging11010013

**Published:** 2025-01-07

**Authors:** Krittapat Onthuam, Norrawee Charnpinyo, Kornrapee Suthicharoenpanich, Supphaset Engphaiboon, Punnarai Siricharoen, Ronnapee Chaichaowarat, Chanakarn Suebthawinkul

**Affiliations:** 1 International School of Engineering, Faculty of Engineering, Chulalongkorn University, Bangkok 10330, Thailand; krittapo@stud.ntnu.no (K.O.); 6238098321@alumni.chula.ac.th (N.C.); 6238001421@alumni.chula.ac.th (K.S.); 6238213121@alumni.chula.ac.th (S.E.); ronnapee.c@chula.ac.th (R.C.); 2 Department of Marine Technology, Norwegian University of Science and Technology, 7491 Trondheim, Norway; 3 Department of Computer Engineering, Faculty of Engineering, Chulalongkorn University, Bangkok 10330, Thailand; 4 Department of Obstetrics and Gynecology, Faculty of Medicine, Chulalongkorn University, Bangkok 10330, Thailand

**Keywords:** deep learning, embryo image, embryo morphology, CNNs, GANs, in vitro fertilization

## Abstract

The current process of embryo selection in in vitro fertilization is based on morphological criteria; embryos are manually evaluated by embryologists under subjective assessment. In this study, a deep learning-based pipeline was developed to classify the viability of embryos using combined inputs, including microscopic images of embryos and additional features, such as patient age and developed pseudo-features, including a continuous interpretation of Istanbul grading scores by predicting the embryo stage, inner cell mass, and trophectoderm. For viability prediction, convolution-based transferred learning models were employed, multiple pretrained models were compared, and image preprocessing techniques and hyperparameter optimization via Optuna were utilized. In addition, a custom weight was trained using a self-supervised learning framework known as the Simple Framework for Contrastive Learning of Visual Representations (SimCLR) in cooperation with generated images using generative adversarial networks (GANs). The best model was developed from the EfficientNet-B0 model using preprocessed images combined with pseudo-features generated using separate EfficientNet-B0 models, and optimized by Optuna to tune the hyperparameters of the models. The designed model’s F1 score, accuracy, sensitivity, and area under curve (AUC) were 65.02%, 69.04%, 56.76%, and 66.98%, respectively. This study also showed an advantage in accuracy and a similar AUC when compared with the recent ensemble method.

## 1. Introduction

In vitro fertilization (IVF) is an assisted reproduction process where an egg is fertilized with sperm in vitro. The fertilized egg, or embryo, is then transferred to the patient’s uterus. With traditional embryo selection methods, the success rate for IVF is insufficient at 20–30% [1,2]. To increase the possibility of pregnancy, the embryo selected to be transferred should have certain characteristics that exhibit high fitness. Currently, embryo selection is performed manually by embryologists based on morphological features, such as the Istanbul grading system [3], which is a numerical version of the Gardner scale [4], and is used to assess embryo quality based on the stage of development, quality of the inner cell mass (ICM), and trophectoderm (TE). Good-grade embryos increase the probability of successful pregnancy and are prioritized to be transferred to the patient’s uterus [5,6]. Although the assessment process is handled manually, the grades of the assessed embryo may be subjective because they depend on the perspective and experience of the evaluating embryologist. In addition, embryos with similar morphological grading require further evaluations to be prioritized, and the best one can then be selected for transfer.

To assist embryologists in embryo selection, machine learning and deep learning techniques have been previously explored to solve various seemingly impossible challenges. Deep learning models, primarily convolutional neural networks (CNNs), have been utilized in assessing embryo viability [7,8,9]. Chen et al. [9] created an autonomous embryo grading system based on the Gardner grading scale, which grades the embryo according to its blastocyst development, ICM, and TE. A total of 171,239 images were used in this work. The images were resized and then fed into the ResNet-50 CNN model, which achieved 75.36% accuracy.

Several studies have developed deep learning models using data from the state-of-the-art closed time-lapse incubator, which provides extensive morphological and morphokinetic results, including video data [10,11,12,13,14,15]. However, static images based on light microscopy remain the standard for IVF laboratories for evaluating embryo quality and viability, particularly in developing countries where access to advanced and high-cost technologies is limited, resulting in a relatively small dataset.

Studies have attempted to utilize conventional CNNs with small datasets in embryo images. Thirumalaraju et al. [16] evaluated several CNN structures for distinguishing blastocysts and non-blastocysts based on their morphological qualities using a dataset of 1188 images. Xception was the best-performing architecture, which achieved 90.48% accuracy on a test set of 742 images. Previously [17], we predicted embryo development using several models based on 1099 images and imbalance datasets with Istanbul grading scores. EfficientNet-B0 was the best model, with 65.0% accuracy. However, other factors, such as the patient’s age during egg retrieval, embryo ploidy status, uterine factors, and embryo transfer procedures, can also affect embryo viability.

The efficiency of a small dataset can also be improved using a generative adversarial network (GAN) [18], which increases the size of the dataset by generating more images. Divaranus et al. [19] used time-lapse images to generate embryo images in various states from 1 to 4 cells. Consequently, the deep neural network demonstrated a 12.3% misclassification rate. In addition, no significant variations were observed between the genuine and synthetic embryo images. Therefore, images produced by GANs can make the small real-image dataset viable for conventional CNNs.

Despite extensive studies on embryo images using CNNs, several studies have evaluated an ensemble learning method, which combines several confidences of models and improves the performance of the embryo image model [20,21,22]. Vermilyea et al. [23] developed an ensemble CNN model to predict human embryo viability using a dataset of 5282 (3892 training and 390 validation images) light microscopic images of a single day 5 embryo in a pilot study to determine what techniques should be used. The model was also improved in a pivotal study using a dataset of 3604 images (1744 training and 193 validation images). After preprocessing, the images were combined into an ensemble model that consisted of eight deep learning models, such as the ResNet-152 and DenseNet-161 architectures. The ensemble model achieved an overall accuracy of 64.3%. Recent works have also studied the multi-input ensemble method [24,25]. Liu et al. [24] statistically examined the importance of each additional feature. The work achieved a top area under receiver operating characteristic (ROC) curve (AUC) of 0.77. The work also discovered that the most impactful features are extracellular. Miyagi et al., though, constructed a pipeline that utilizes intercellular features and achieved a similar result, with a maximum AUC of 0.74. The work also states that the AUC is affected by the patient’s maternal age interval.

However, embryo image analysis may be more conclusive if additional features are used. As embryo viability is commonly considered from the combined perceptions received from Istanbul scores, such scores could also be predicted using CNNs. The predictions of the Istanbul grading score can improve the performance of the model by providing extra inputs for consideration. A combined input model is feasible. Although there are varying opinions about the significance of the Istanbul score in model development, several works found that these features are related to successful embryo development [2,26,27,28]. However, our method addresses this by standardizing these features using automatically extracted data, ensuring consistency. As a continuation of our previous work, our study aimed to develop a deep learning model-based pipeline that efficiently classifies viable and nonviable day 5 embryos from a combined input of light microscopic images and other associated factors using an imbalanced and relatively small dataset of embryo images. Our system requires a microscopic image of a day 5 embryo along with the patient’s age. The image is then extracted automatically for its continuous levels of blastocyst development, ICM, and TE. These features are trained from Istanbul grading scores labeled by embryologists but are optimally adjusted for the prediction model to ensure the consistency of these additional features, reducing variability introduced by future human interpretation and enhancing the performance of the prediction model. Furthermore, this study evaluated the performance of a model trained using day 5 embryo data generated by a GAN. This paper is organized as follows: Section 2 specifies the data, explains the model training process, and details the configurations experimented with to gradually improve the model; Section 3 presents and compares the performance of the model between each configuration; Section 4 discusses the prediction results obtained from the model and improvement possibilities; and Section 5 concludes the key findings.

## 2. Materials and Methods

### 2.1. Dataset

Data were obtained from patients who had undergone IVF at the Reproductive Biology Unit, King Chulalongkorn Memorial Hospital, from 2018 to 2022. Data were limited to patients who received a single day 5 embryo transfer, and the endpoint was clinical pregnancy indicated by fetal heartbeat at first transvaginal ultrasonography at 6–10 weeks. Each patient’s record contained the image of the embryo at day 5, the patient’s age at the time of egg retrieval, Istanbul grading scores (stage, ICM, and TE) by the embryologists, and implantation results classified as pregnant and non-pregnant. The details are shown in Table 1. The records are entered into the model as inputs. Some records may not contain all the aforementioned data. Embryo images were acquired taken via light microscopy or scanning.

This study was approved by the Institutional Review Board, Faculty of Medicine, Chulalongkorn University (IRB No. 686/65). The Thai Clinical Trials Registry identification number is TCTR20230130002. The need for informed consent was waived owing to the retrospective study design and the anonymous use of clinical data.

### 2.2. Embryo Pregnancy Prediction

**Figure 1:**
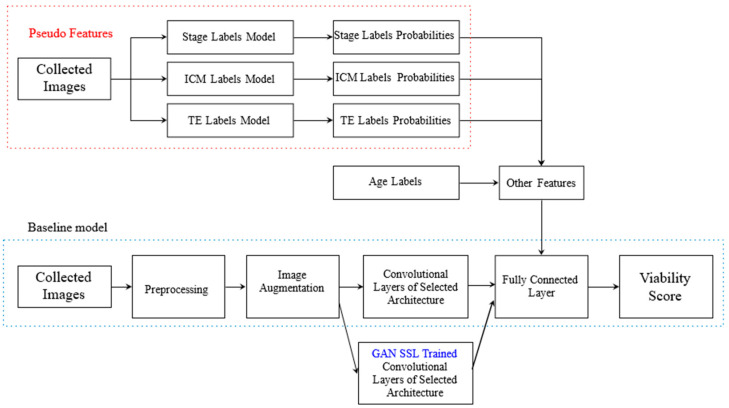
Proposed pipeline of embryo pregnancy prediction using combined inputs. GAN, generative adversarial networks; ICM, inner cell mass; TE, trophectoderm.

Our proposed architecture of embryo pregnancy prediction is shown in Figure 1. The baseline model received a light microscopic image input of the embryo, uninformative pixels were removed, and standardization was performed in preprocessing. To increase the generalizability of the model, augmentation was employed, including flipping, rotation, and slight shifting. The features of each embryo were then extracted using CNNs, followed by a fully connected layer to output the confidence score of viability. The proposed architecture combined the baseline model and probabilities of the three pseudo-features, including stage, ICM, and TE, which were separately predicted by additional models. The pregnancy outcome of an embryo was, therefore, determined by a concatenation of the convolutional features of the images of the embryo, pseudo-feature values, and patient age.

### 2.3. Preprocessing

**Figure 2:**
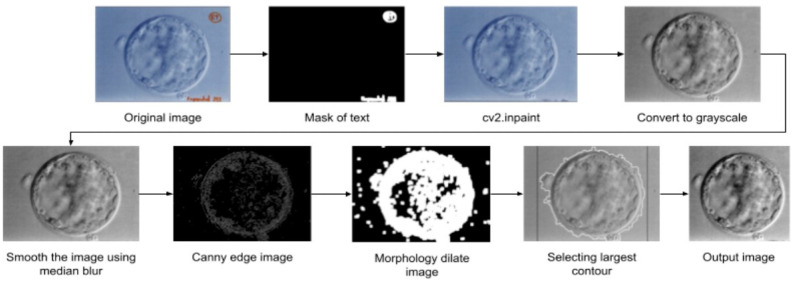
Scanned embryo image preprocessing diagram.

Some of the scanned embryo images had uninformative content, such as hand-written annotations. To prevent the model from losing its focus, these annotations were removed. Figure 2 illustrates the overall preprocessing for scanned images. First, thresholding was used to mask the letters in the image. The annotations are significantly higher in intensity as compared to the embryo visual contents. Therefore, the threshold masking does not have any negative effects on the embryo’s visual quality. Then, a process of inpainting [28] was applied to remove letters by replacing the color of the pixel of the annotation with that of its neighboring pixel. After that, median blur was applied to smooth out the image because of salt and pepper noise. Then, edges were detected using Canny, and dilation was applied to the edge image to connect small edges. Thereafter, the largest contour in the image was detected. Then, the image was cropped based on the contour and resized to 224 × 224 resolution.

### 2.4. Backbone Architecture Selection

The selection of the backbone model is a crucial choice. Several state-of-the-art and baseline models were experimentally selected and trained on our dataset using binary cross entropy loss (BCE) or focal loss, a modified version of BCE that is penalized more on incorrect predictions. We selected a combination of CNN and transformer-based methods, including ResNet-34 [29], EfficientNet-B0 [30], CoAtNet-2 [31], Xception [32], and ViT-Tiny-S16 [33]. Fundamentally, the ResNet model uses skip connections, which allow the information to skip past one or more layers within the network. These connections help the network focus on learning residual functions, which are essentially the discrepancies between a layer’s input and output. EfficientNet uses a combination of model scaling, and neural architecture search is used to achieve the ideal balance between the size and accuracy of the model. Xception is convolutional networks that contain variable sizes of filters, with depth-wise separable convolutions that improve the computational efficiency of the model. EfficientNet-B0 is the base version from which other versions of CoAtNet-2, which is the fusion of convolution and transformer, leverages the strengths of both architectures to enhance performance. The vision transformer (ViT) allows the model to capture complex relationships between patches, improving its ability to understand and categorize visual content effectively.

### 2.5. Pseudo-Features

Pseudo-features were created to replace the original embryo grading scores given by embryologists. These are non-discrete parameters representing Istanbul scores. Pseudo-features consist of three parameters, each of which represents the probabilities of embryo labels in stage, ICM, and TE. Three additional models were separately created to predict each parameter, and an experiment was conducted to obtain the optimal pseudo-feature-predicting model architecture.

### 2.6. Custom Weight Using Simple Framework for Contrastive Learning of Visual Representations (SimCLR)

**Figure 3:**
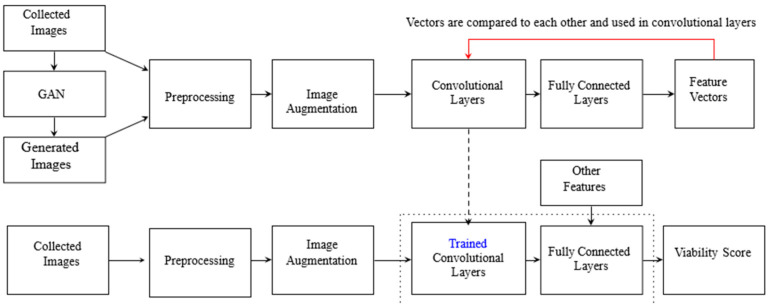
Proposed structure of the model trained using Simple Framework for Contrastive Learning of Visual Representations (SimCLR) on original and generative adversarial networks (GAN)-generated images.

Given the limited dataset, possibilities to generate an embryo dataset based on the available dataset were explored. In addition to the baseline model and pseudo-feature extraction models, a custom weight was developed using GAN-generated embryo images obtained by training original images on a GAN. A GAN is an artificial neural network designed for creating new data that resemble the data used in its training, and it is applicable in fields such as image generation. GANs are composed of a generator, which produces the data, and a discriminator, which evaluates its authenticity. In this study, generative images from a conditional GAN (CGAN) [34], which generates new images based on given labels, e.g., viable or non-viable, and a Wasserstein GAN (WGAN) [35], which is trained to approximate the distance between the real and generated images, were combined. The quality of the GAN-generated image is evaluated by visual inspection and aligns with the characteristics of the original embryo images. The generative images were combined with the original images and then used for training in SimCLR (Figure 3) [36], which is a self-supervised learning approach designed to capture valuable data representations by increasing the similarity of the enhanced versions of the same image while decreasing the similarity of the enhanced versions of different images. This method employs a contrastive loss function that helps position similar items closer together and dissimilar items farther apart in the embedding space.

### 2.7. Hyperparameter Optimization

To obtain a more accurate prediction, a custom prediction threshold was calculated based on the uneven pregnancy class ratio using the same method as our previous work [17]. The model hyperparameters were optimized using Optuna [37], a Python library that provides an automated framework for hyperparameter optimizations by finding a combination of hyperparameters that yields the best performance. It works by creating a search space of hyperparameters for the model, which defines the range and distribution of values that each hyperparameter can take. Optuna uses a combination of Bayesian optimization and pruning techniques to explore the search space efficiently. Bayesian optimization is a probabilistic approach that models the performance of a model as a function of its hyperparameters. The model’s performance was used to update the probability distribution of the hyperparameters. Pruning techniques were used to discard unpromising hyperparameters and avoid wasting computational resources on training them.

## 3. Results

This study included a total of 1194 images with 371 pregnant and 823 non-pregnant images. The overall clinical pregnancy rate was 31.07%. The patient age interval in the dataset was between 25 and 50 years, with a mean age of 37.8 ± 4.0 years. The summary of the dataset is shown in Table 1. The data were split into training, validation, and testing sets with a ratio of 60%, 20%, and 20%, respectively. To guarantee that the minority and majority class distributions in each split data set are identical, the stratified random sampling technique was employed.

Comparative experiment studies were conducted to analyze the performance of each component in our proposed pipeline for classifying embryo images. The following experiments were conducted consecutively:

1. Selecting a baseline model: Each baseline model was trained on the dataset. The backbone of the best-performing model was selected for the next experiment and used for generating pseudo-features.
2. Evaluating the integration of pseudo-features: Pseudo-features were created using the optimal model from a previous experiment. The features were then combined with the baseline model to create a pipeline according to Figure 3. The same pipeline using the real Istanbul grading feature was developed in parallel and compared with the pseudo-features. In addition, the patient age label was scaled in such a way that the mean value and standard deviation were zero.
3. Optimization using Optuna: The best model from Experiment 2 was then optimized using Optuna [29]. The performances of the model before and after optimization were compared.
4. Comparison of the best baseline with the custom weight: A custom-weight model was developed using the same method as used in Experiments 2 and 3. To obtain the best model, the model was compared with the model in Experiment 3.

### 3.1. Evaluation Metrics

Several metrics were recorded for each model trained to measure model performance. These metrics include the average sensitivity, specificity, accuracy, area under curve (AUC) of the receiver operating characteristic curve, and F1 score. Equations (1)–(4) show the calculations for each metric, where TN, FN, TP, and FP are the true negatives, false negatives, true positives, and false positives, respectively. Sensitivity and specificity are important metrics for medical applications; however, low sensitivity may result in missing viable embryos, whereas low specificity could lead to unnecessary transfers of non-viable embryos. Thus, the F1 score was primarily prioritized in our experiments. The AUC is also used to measure the area under the true positive rate (Sensitivity) and the false positive rate (1−Specificity) to quantify the ability of the model to distinguish between pregnant and non-pregnant classes. In addition, a confusion matrix was used to visualize the overall performance of the model along with a kernel density plot on each Istanbul grading score to illustrate the correspondence between the predictions by the model and embryologists.

(1) $$Accuracy=\frac{TN+TP}{TN+TP+FN+FP}$$

(2) $$Sensitivity=\frac{TP}{TP+FN}$$

(3) $$Specificity=\frac{TN}{TN+FP}$$

(4) $$F1-Score=\frac{2TP}{2TP+FN+FP}$$

### 3.2. Baseline Model Selection

ResNet-34, EfficientNet-B0, CoAtNet-2, Xception, and ViT-Tiny-S16 pretrained models were tested and compared using BCE loss. The evaluation metrics for each model are shown in Table 2. The best-performing model was EfficientNet-B0 with 70.00% sensitivity, 62.73% accuracy, an AUC of 67.2%, and an F1 score of 61.45%.

### 3.3. Pseudo-Features Integration

As mentioned in Section 1 EfficientNet-B0 achieved the best performance. The architecture was, therefore, used to implement the pseudo-feature models. The performance of the combined model using pseudo-features was then compared with the model with labeled features. The result of this change is shown in Table 3. The model using the pseudo-features of stage, ICM, and TE had significant advantages, with 4.24% specificity, 2.41% accuracy, and an F1 score of 1.88%, compared to the manually labeled features and was slightly higher than the baseline model without additional features.

### 3.4. Hyperparameter Optimization Using Optuna and Custom Weights

Optuna is an automated framework that was used to optimize the hyperparameters of the EfficientNet-B0 model with pseudo-features. The hyperparameters selected by Optuna were as follows: learning rate = 1 × 10*^−^*^4^, L2 regularization strength = 1 × 10*^−^*^5^, dropout rate = 0.4, number of nodes in the fully connected layer right before the output layer = 10 (2 image nodes, 8 additional features nodes), and loss function as focal loss. Simultaneously, a custom-weight model based on the EfficientNet-B0 model was trained using SimCLR with GAN-generated images. Table 4 compares the performance of both newly developed models. The performance of the pseudo-feature model was significantly improved, with increases of 2.71% in sensitivity, 3.55% in accuracy, 3.98% on the AUC, and 3.30% on the F1 score.

## 4. Discussion

From our experiments, the best backbone model was EfficientNet-B0 with preprocessed images, scaled age, and pseudo-features as inputs. The scalability quality of EfficientNet, which prevents overfitting in small datasets, could contribute to its top performance. In addition, EfficientNet-B0 outperforms CoAtNet and ViT due to its lightweight design, making it less prone to overfitting and better at adapting to generalization with limited data. Preprocessing removes noises and red herrings, allowing the model to focus on distinctive regions. The pseudo-features assisted the model in classifying the images better because they are continuous values, which are more suitable for neural networks, compared with categorical values. In addition, the hyperparameter tuning process significantly affected the performance of the models. Their performance improved remarkably from 65.59% to 69.04% in accuracy and from 61.72% to 65.02% in the F1 score. The learning rate and number of nodes were key factors assisting the models to converge. Our experiments also showed that the self-supervised model from the GAN dataset was ineffective at improving the pipeline performance. This may result from the low quality of the generated images in terms of their lack of variety and realness. Furthermore, the number of images created for weight development was considerably small. The pipeline requires further exploration of the optimal procedures for generating day 5 embryo images using a GAN with an optimal quality and quantity. Additionally, our GAN could suffer from a mode collapse problem and limited variations in generated images. An alternative approach for generating images that is less prone to limited variations, such as diffusion-based models, could be explored and further evaluated.

**Figure 4:**
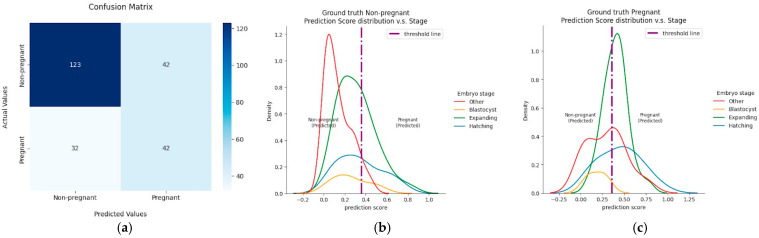
Model performance summary: (**a**) confusion matrix of best model on test dataset, (**b**) prediction score distribution (non-pregnant), and (**c**) prediction score distribution (pregnant).

Figure 4a shows the confusion matrix of the best model. The model performed well in predicting non-pregnant states but not in pregnancy prediction. This is evident in the number of false negatives: the number of embryos with a ground truth of pregnant that were predicted to be non-pregnant. This is a significant proportion of correct pregnancy prediction and led to the tendency for the prediction score of the embryos with a pregnant ground truth to be lower than the threshold (Figure 4c). This could be due to the dataset including large amounts of non-pregnant images. Thus, the model is likely to predict a non-pregnant state, which corresponds to its high specificity.

The imbalanced dataset severely impacted the model’s performance and sensitivity. For the viable class (minority), sensitivity is reduced because the model tends to favor the majority class (non-viable class). Figure 4 shows the distribution of prediction scores according to the embryo stage with non-pregnant (Figure 4b) and pregnant (Figure 4c) ground truths. The prediction score tended to be over the threshold when the embryo was in the expanding or hatching state. The performance of the model was observed to be aligned with how embryologists observe the embryos. However, the rationality of the prediction was unclear, as it was the result of several model predictions. To concretely explain the prediction, model interpretation could be further done using CNN-related methods, such as a gradient classification map. In future works, the prediction score could be applied to exclusively select which embryo from the patient to be transferred.

The accuracy of the model was approximately 70%, which is acceptable in IVF standards. To evaluate the performance of the pipeline, it was compared with another work using the ensemble method [23]. Our best model showed an advantage in accuracy and specificity but lagged significantly with a sensitivity of 14.56%. This could result from our dataset having significantly more non-pregnant images than pregnant images, whereas the dataset in [24] had similar amounts of both classes. However, more experiments on ensemble methods could be performed to find the optimal method for integrating each feature. Furthermore, our work achieved a similar AUC to [25] when using only a blastocyst image as an input. The additional extracellular features in [25] also significantly improved the AUC by more than was seen in our study. However, our pseudo-feature is entirely predicted from the input image and focused on the intercellular feature. The work in [26] also showed the significance of these features in model development. The work also utilized the variation of AUC values with maternal age, which is a significant feature for the model [17,25,26]. Although we achieved a lower sensitivity and specificity than [26], our model requires only light microscopic images as an input. This could lead to simpler implementation and satisfy our aim to develop an assistant pipeline for embryologists.

The overall model performance may be improved using more varied data. In this study, only day 5 embryo images were utilized, because it is a common day of pregnancy consideration. However, as shown in Table 1, this dataset comprised approximately 32% of the images of day 5 embryos that are yet to reach the blastocyst stage, which are largely with a non-pregnant ground truth. This significant imbalance could lead to the tendency towards non-pregnant predictions of the model. Therefore, the ratio of these images can be reduced or completely removed to balance the dataset and improve model sensitivity. In addition, exclusive experiments to increase model sensitivity could be conducted. This includes comparing the model using different loss functions, such as weigh cross entropy with a higher weight on the viable embryos. GANs could also be used exclusively to generate more viable embryo images in an attempt to balance the dataset. Different models, such as decision trees, could also be explored.

In addition, models in the pipeline have utilized the data collected by continuous monitoring of the development of each embryo, such as using time-lapse images. Other types of data, such as morphokinetic parameters obtained from time-lapse images or video data, could yield a significant improvement in terms of image quantity and provide more insights into the model. Other factors, such as the semen quality and male factors, embryo ploidy status, uterine factors, and embryo transfer procedures, can also affect a successful pregnancy. Furthermore, the endpoint of pregnancy as measured by a fetal heartbeat may not determine a successful live birth.

This study was performed using retrospectively collected data; thus, it was not intended for deep learning model development. Although time-lapses and video data are more suitable, their access is limited in developing countries. Therefore, exploring the optimal method on conventionally collected data is necessary. As a strength, this study combines small image information with the grading scores by the embryologists, which pioneers the methodology of applying artificial intelligence to medical image analysis. It aims to create an assistant system for embryologists using conventionally collected data, which could allow for further related studies in developing countries.

## 5. Conclusions

This study introduces a CNN-based combined input model pipeline to assist embryologists in classifying embryos to increase IVF success. The model predicts the probability of the implantation of each embryo using the combined inputs of light microscopic images, patient age, and pseudo-features according to the Istanbul grading system, including embryo stage, ICM, and TE, predicted by EfficientNet-B0. Moreover, this study compared the performance of conventional CNNs with our implemented network using GAN and SimCLR to tackle the problem of the relatively small dataset. Our experiments showed that the EfficientNet-B0 model uses preprocessed images, age, and generated pseudo-features as inputs. The hyperparameters of the models were also tuned using Optuna, which yielded an F1 score of 65.02%, accuracy of 69.04%, sensitivity of 56.76%, specificity of 74.55%, and AUC of 66.98%. The proposed model can be used by embryologists to prioritize and select the best embryo for transfer. The performance of the model could be improved in image input in both quantity and quality. The proposed model is a first step in paving the way to establish better tools for assisting embryologists in selecting and prioritizing high-potential embryos. In combination with gradient-weighted class activation map technology, this model can be applied as a practice tool for embryologists prior to actual embryo selection. Additionally, this model can be a helpful tool in the situation of disagreement among embryologists. The web-based software application, integrated with the EfficientNet-B0 model, can be applied in IVF clinics across the country and globally.

## Figures and Tables

**Table 1:** Summary of datasets classified by pregnancy outcomes.

Factors	All	Pregnant	Non-Pregnant
	N (%)	N (%)	N (%)
Age (years, mean *±* SD)	37.8 *±* 4.0	36.8 *±* 3.7	38.2 *±* 4.0
Stage of Embryo			
Cavitate/Early Cavitate	379 (31.74)	77 (20.75)	302 (36.70)
Early Blastocyst	44 (3.66)	18 (4.85)	26 (3.16)
Blastocyst	74 (6.20)	26 (7.00)	48 (5.83)
Expanded	476 (39.87)	151 (40.70)	325 (39.49)
Hatching/Hatched	221 (18.51)	99 (26.68)	122 (14.82)
Inner Cell Mass (ICM)			
Good	366 (30.65)	133 (35.85)	233 (28.31)
Fair	295 (24.71)	111 (29.92)	184 (22.36)
Poor	110 (9.21)	32 (8.63)	78 (9.48)
No Label	423 (35.43)	95 (25.61)	328 (39.85)
Trophectoderm (TE)			
Good	205 (17.17)	91 (24.53)	114 (13.85)
Fair	395 (33.08)	146 (39.35)	249 (30.26)
Poor	171 (14.32)	39 (10.51)	132 (16.04)
No Label	423 (35.43)	95 (25.61)	328 (39.85)

**Table 2:** Performance of pretrained models using only images as input and binary cross entropy (BCE) loss.

Model	Metrics				
	F1-Score	Sensitivity	Specificity	Accuracy	AUC *
ResNet-34	51.03%	57.14%	50.00%	52.27%	60.0%
EfficientNet-B0	61.45%	70.00%	59.33%	62.73%	67.2%
CoAtNet-2	60.34%	60.00%	64.00%	62.73%	65.0%
Xception	56.55%	50.00%	64.67%	60.00%	62.3%
ViT-Tiny-S16	56.56%	44.59%	69.33%	61.36%	64.1%

* AUC, area under curve.

**Table 3:** Performance of EfficientNet-B0 model with preprocessing using real features and pseudo-features.

Model	Metrics				
	F1-Score	Sensitivity	Specificity	Accuracy	AUC *
EfficientNet-B0	61.45%	70.00%	59.33%	62.73%	67.2%
EfficientNet-B0 + Three Labelled Features (Stage, ICM *, TE *) + Age	59.84%	55.41%	66.67%	63.18%	64.33%
EfficientNet-B0 + Three Pseudo Features (Stage, ICM, TE) + Age	61.72%	54.05%	70.91%	65.59%	63.01%

* ICM, inner cell mass; TE, trophectoderm; AUC, area under curve.

**Table 4:** Results of EfficientNet-B0 model using pseudo-features with and without Optuna.

	Metrics				
	F1-Score	Sensitivity	Specificity	Accuracy	AUC *
Pseudo Features	61.72%	54.05%	70.91%	65.69%	63.01%
Pseudo Features + Optuna	65.02%	56.76%	74.55%	69.04%	66.98%
Custom-weight model trained using GAN * images	62.54%	52.70%	73.33%	66.95%	64.78%

* GAN, generative adversarial network; AUC, area under curve.

## Data Availability

All original data in this publication are available upon reasonable request to the corresponding author.
